# Relationship between liver fat, pancreatic fat, and new-onset type 2 diabetes mellitus in patients with metabolic dysfunction-associated fatty liver disease

**DOI:** 10.1007/s00592-025-02501-7

**Published:** 2025-04-19

**Authors:** Huanjia Qu, Lingling Zhou, Dong Tang, Qiuling Zhang, Pu Yang, Boyi Yang, Junping Shi

**Affiliations:** 1 Department of Endocrinology, Affiliated Hospital of Hangzhou Normal University, Hangzhou, 310015 China; 2 Department of Radiology, Affiliated Hospital of Hangzhou Normal University, Hangzhou, 310015 China; 3 Department of Endocrinology, Hangzhou Normal University, Hangzhou, 310000 China; 4 Department of Metabolic Disease Center, Affiliated Hospital of Hangzhou Normal University, Hangzhou, 310015 China

**Keywords:** Metabolic dysfunction-associated fatty liver disease, Type 2 diabetes mellitus, Liver fat fraction, Pancreatic fat fraction, Ectopic fat deposition, MRI

## Abstract

**Purpose:**

Type 2 diabetes mellitus (T2DM) is associated with ectopic fat deposition, especially in the liver and pancreas. Therefore, this study aimed to evaluate the relationship between liver fat fraction (LFF), pancreatic fat fraction (PFF), and new-onset T2DM in metabolic dysfunction-associated fatty liver disease (MAFLD) by magnetic resonance imaging (MRI).

**Methods:**

This is a retrospective study of patients with MAFLD who underwent abdominal MRI between 2022 and July 2024. LFF and PFF were measured using an axial multi-echo Dixon-based sequence. All participants underwent routine medical history, anthropometric measurements, and laboratory tests. Multivariable stepwise selection models were constructed to predict PFF and T2DM status based on variables of clinical interest.

**Results:**

This study included 80 MAFLD patients with 40 untreated new-onset T2DM and 40 non-T2DM controls. LFF, PFF, and homeostasis model assessment of insulin resistance (HOMA-IR) index were higher in the T2DM group than in the control group. In the new-onset T2DM group, PFF was linearly positively correlated with LFF (*r*_*s*_ = 0.321, *P* = 0.04) and HOMA-IR (*r*_*s*_ = 0.350, *P* = 0.03). After adjustment for several metabolic variables, PFF remained an independent risk factor for incident T2DM in MAFLD patients (all *P* < 0.05). The area under the receiver operating characteristic curve for PFF and LFF to predict T2DM was 0.889 and 0.633 (*P* < 0.001 and *P* = 0.03), respectively.

**Conclusion:**

In MAFLD patients, PFF, and LFF play a prominent role in new-onset T2DM with high predictive and diagnostic value.

## Introduction

Obesity leads to adipocyte dysfunction and increased levels of free fatty acids (FFAs). Excessive supply of FFAs and lipids beyond adipose tissue adaptation leads to adipocyte insulin resistance (IR) and ectopic fat deposition in organs, such as the heart, liver, and pancreas. Metabolic-associated fatty liver disease (MAFLD), formerly known as non-alcoholic fatty liver disease (NAFLD), is a multisystemic metabolic disorder characterized by the accumulation of fat in the liver during obesity, type 2 diabetes mellitus (T2DM) or other metabolic disorders [1]. It is now recognized as the most common cause of chronic liver disease worldwide, affecting more than 30% of the global population [2].

Hepatic steatosis is a major contributor to the development of IR and inflammatory complications [3]. T2DM is a metabolic disease characterized by chronic hyperglycemia associated with IR and impaired insulin secretion [4]. The prevalence of diabetes continues to rise worldwide; it is estimated that by 2030, approximately 366 million people will have diabetes, of whom more than 90% will have T2DM [5], with a higher prevalence of T2DM among obese adults [6].

Therefore, early detection of incipient T2DM is clinically important, especially in patients with MAFLD. The onset and progression of T2DM are associated with systemic fat distribution and localized fat deposition. The pancreas is another important metabolic organ, and intrapancreatic fat deposition usually occurs in T2DM. However, its pathophysiological effects remain unclear, and its impact on metabolism, IR, and pancreatic islet cell function has not been fully investigated [7, 8]. As a result, the relationship between pancreatic fat content and T2DM remains controversial, and there are specifically few studies on pancreatic fat content and T2DM in the MAFLD patients. Several studies have shown that individuals with T2DM have significantly higher intrapancreatic fat deposition than healthy individuals [9], which may lead to IR, β-cell dysfunction, hyperglycemia, and other related diabetes complications [10–12]. However,there are different views among a portion of the population [13–15]. These conflicting conclusions may be due to differences in study populations, ethnicity, disease status, and quantitative techniques used. Currently there are various techniques to detect pancreatic steatosis, including histology, ultrasound, computed tomography (CT), and magnetic resonance imaging (MRI), but there is no validated enzymatic or imaging technique available that can easily and accurately assess pancreatic steatosis. The use of chemical shift-encoded MRI for pancreatic fat fraction (PFF) detection has been validated based on histology and found to correlate almost perfectly with fat content in the phantom [16]. Therefore, MRI has become the most natural imaging modality for non-invasive quantification of PFF in humans [17, 18].

In this study, we included MAFLD patients diagnosed by MRI as the study population to investigate the relationship among liver fat fraction (LFF), PFF, metabolic indices related to pancreatic islet cells, and new-onset T2DM, which can provide a reference for clinical evaluation and decision-making.

## Methods

### Study population

This study was performed according to the principles in the Declaration of Helsinki and approved by the ethics board of The Affiliated Hospital of Hangzhou Normal University (Hangzhou, China) [approval number: 2022-(E2)-HS-146]. Patients with MAFLD diagnosed at The Affiliated Hospital of Hangzhou Normal University from June 2022 to June 2024 were included in this study. All participants signed informed consent to participate in the study. The inclusion criteria were as follows: (1) MAFLD diagnosed based on magnetic resonance imaging-proton density fat fraction (MRI-PDFF) [19] and (2) T2DM diagnosed following the World Health Organization (WHO) criteria. Untreated T2DM included newly diagnosed T2DM and previously diagnosed, non-treated T2DM (diabetes duration ≤ 2 years). The exclusion criteria were as follows: (1) age < 14 years; (2) other types of diabetes (*e.g*., type 1 diabetes mellitus, gestational diabetes mellitus, and other specific types); (3) patients with liver diseases caused by drugs, viral hepatitis, and other causes, excluding alcohol and those with a previous history of myocardial infarction, cerebral hemorrhage, and stress conditions in the last 3 months; (4) pregnant and lactating women; (5) MRI contraindications (metallic implants, claustrophobia, body circumference exceeding the magnet bore size).

## Study methods

### Laboratory and demographic data

All participants underwent a detailed medical history collection and physical examination including sex, age, and body mass index (BMI). The next day after admission, venous blood was collected from patients on an empty stomach in the early morning after nighttime fasting (12 h). Fasting plasma glucose (FPG), alkaline phosphatase (ALP), alanine aminotransferase (ALT), gamma-glutamyl transferase (GGT), serum uric acid (SUA), total cholesterol (TC), low-density lipoprotein cholesterol (LDL-C), high-density lipoprotein cholesterol (HDL-C), and triglyceride (TG) were measured in serum using an AU5800 automatic biochemistry analyzer (Beckman Coulter, Brea, CA, USA). The level of glycosylated hemoglobin A1c (HbA1c) was determined using a Tosoh G8 HPLC Automated Glycohemoglobin Analyzer (Tosoh Bioscience, Tokyo, Japan). Homeostasis model assessment of insulin resistance (HOMA-IR) index was calculated using the formula HOMA-IR = [FPG(mmol/L) × fasting insulin (µU/mL)]/22.5.

### MRI acquisition and analysis

All patients were required to fast for at least 10 h before undergoing an MRI scan examination performed on a Magnetom Avanto 1.5-T system (Siemens, Erlangen, Germany) using a 16-channel phased-array coil. A single breath-hold acquisition provided a multi-echo chemical shift–encoded gradient echo sequence. Image postprocessing was performed with a fitting algorithm to calculate liver and pancreas fat content. Based on recent studies, we used a 5.5% LFF threshold to define any degree of steatosis. The LFF and PFF were obtained with manual delimitation by a single experienced radiologist, placing three regions of interest (ROIs) of the liver and three drawn on the head, body, and tail of the pancreas, avoiding the major vessel, pancreatic duct, adjacent visceral fat, and artifacts. Image analysts were blinded to clinical and histological data.

### Statistical analysis

Data were analyzed using the SPSS 29.0 software (IBM Corporation, Armonk, NY, USA). Continuous data were tested for normality and homogeneity of variance using the Shapiro–Wilk and Levene tests. Normally distributed data are expressed as the mean ± standard deviation (range). The differences between two groups were evaluated by the t-test, and between multiple groups by one-way analysis of variance (ANOVA). Non-normally distributed data are expressed as the median (interquartile range) and the differences between the two groups were evaluated using the Mann–Whitney U test, and between multiple groups using the Kruskal–Wallis H test. For categorical variables, percentages and χ^2^ tests were used to describe and analyze the data. Pearson or Spearman correlation analysis was used to evaluate the correlation between the PFF and LFF. To determine the association between PFF and metabolic variables, the odds ratios (ORs) and 95% confidence intervals (CIs) were calculated using ordinal logistic regression analysis. The analyses included three models: model 1, which was unadjusted; model 2, which was adjusted for age and sex; model 3, which was adjusted for age, sex, BMI, ALP, SUA, HOMA-IR index and LFF. The area under the receiver operating characteristic (ROC) curve (AUC) was used to establish the diagnostic accuracy of the PFF and LFF to detect T2DM in patients with MAFLD. Youden’s Index was used as the cut-off value in the ROC curve, and was calculated using the following formula: Youden’s Index = sensitivity + specificity − 1. A *P* value < 0.05 was considered to indicate a statistically significant difference.

## Results

### Characteristics of the study participants

This study included 80 patients with MAFLD diagnosed by MRI, divided into two groups, namely the non-T2DM group (*n* = 40) and the new-onset T2DM group (*n* = 40). The baseline clinical characteristics of the stratified study population are shown in Table 1. Compared to the non-T2DM group, the T2DM group tended to have significantly higher ALP, SUA, LFF, FPG, HOMA-IR index, and glycosylated hemoglobin A1c (HbA1c) (all *P* < 0.05). There were no differences in age, BMI, ALT, GGT, TC, TG, LDL-C, and HDL-C between the two groups.

**Table 1:** Characteristics of the new-onset T2DM group and non-T2DM group in MAFLD patients

Variables	All participants (*n* = 80)	T2DM group (*n* = 40)	Control group (*n* = 40)	*P*-value
Age(y)	37.3 ± 11.88	38.53 ± 12.31	36.08 ± 11.44	0.36
Gender(M/F)	64/16	29/11	35/5	0.094
BMI (kg/m^2^)	27.37 (25.82, 29.89)	26.83 (25.57, 29.96)	27.51 (25.84, 30.06)	0.422
ALT (mmol/L)	96 (40.5, 145)	55.5 (30.25, 121)	120.5 (93.25, 172.5)	0.136
GGT (mmol/L)	68.5 (41.25, 115)	61 (36.37, 105.75)	73.5 (42.75, 120)	0.138
ALP (mmol/L)	96.5 (77.25, 110.75)	101.5 (85, 112.5)	86 (71, 99.75)	0.002
FPG (mmol/L)	6.4 (5.37, 8.67)	8.63 (7.79, 13.71)	5.38 (5.07, 5.88)	< 0.001
HbA1c(%)	6.35 (5.6, 10)	9.9 (7.4, 11.7)	5.6 (5.4, 5.8)	< 0.001
HOMA-IR	5.85 (3.88, 7.76)	6.60 (4.82, 8.20)	4.42 (3.02, 7.00)	0.003
TG (mmol/L)	2.24 (1.74, 3.14)	2.72 (1.78, 3.76)	2.1 (1.56, 2.81)	0.062
TC (mmol/L)	5.2 (4.63, 6.07)	5.2 (4.63, 6.31)	5.11 (4.51, 5.86)	0.442
HDL (mmol/L)	1.05 (0.92, 1.22)	1.02 (0.8, 1.22)	1.1 (0.97, 1.23)	0.099
LDL (mmol/L)	3.26 (2.71, 3.85)	3.43 (2.67, 4.08)	3.17 (2.7, 3.74)	0.516
SUA (mmol/L)	415 (339.75, 495)	349.5 (290, 527)	444 (389, 484)	0.039
LFF (%)	15.40 (11.71, 18.6)	16.74 (12.80, 18.92)	13.6 (10.56, 17.80)	0.04
PFF (%)				
Mean	3.58 (2.84, 4.34)	4.20 (3.57, 5.07)	2.86 (2.08, 3.58)	< 0.001
Head	3.30 (2.39,3.84)	3.67 (3.30, 4.59)	2.56 (1.70, 3.31)	0.006
Body	3.57 (2.69, 4.72)	4.72 (3.37, 5.72)	3.16 (1.94, 3.83)	< 0.001
Tail	3.69 (2.43, 4.64)	4.38 (3.58, 6.12)	2.46 (1.62, 4.41)	< 0.001

Values were expressed as mean (SD) medians (quartile interval) or n (%)

T2DM vs. non-T2DM, A *P-*value < 0.05 indicates statistically significant difference

*MAFLD* metabolic dysfunction-associated fatty liver disease, *BMI* body mass index, *WC*: Waist circumference, *ALT* alanine transaminase, *ALP* alkaline phosphatase, *GGT* gamma-glutamyl transferase, *TC* total cholesterol, *TG* triglyceride, *HDL-C* high-density lipoprotein cholesterol, *LDL-C* low-density lipoprotein cholesterol, *FPG* fasting plasma glucose, *HbA1c* glycosylated hemoglobin, *HOMA-IR* homeostasis model assessment of insulin resistance, *SUA* serum uric acid, *LFF* liver fat fraction, *PFF* pancreatic fat fraction

### PFF, LFF and HOMA-IR

**Fig. 1:**
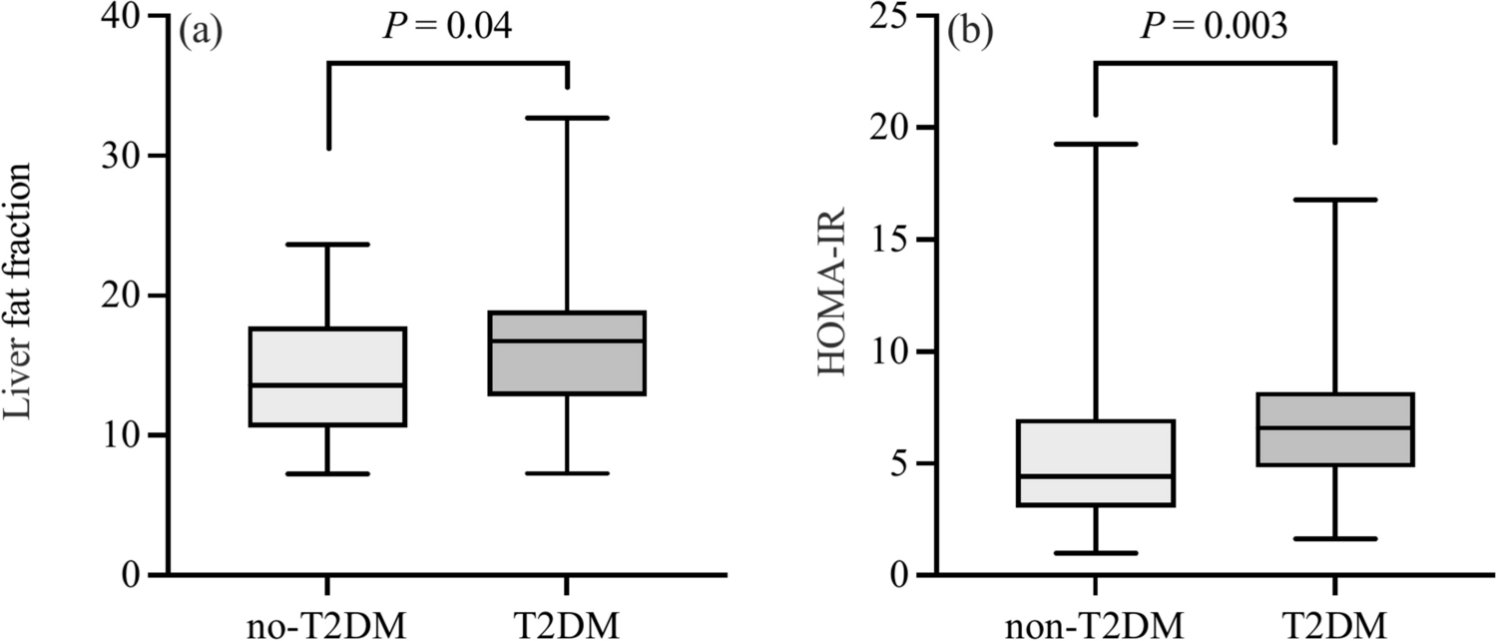
Comparison of the LFF (**a**) and HOMA-IR (**b**) between the non-T2DM group and the new-onset T2DM group in MAFLD patients. A *P-*value < 0.05 indicates statistically significant difference

**Fig. 2:**
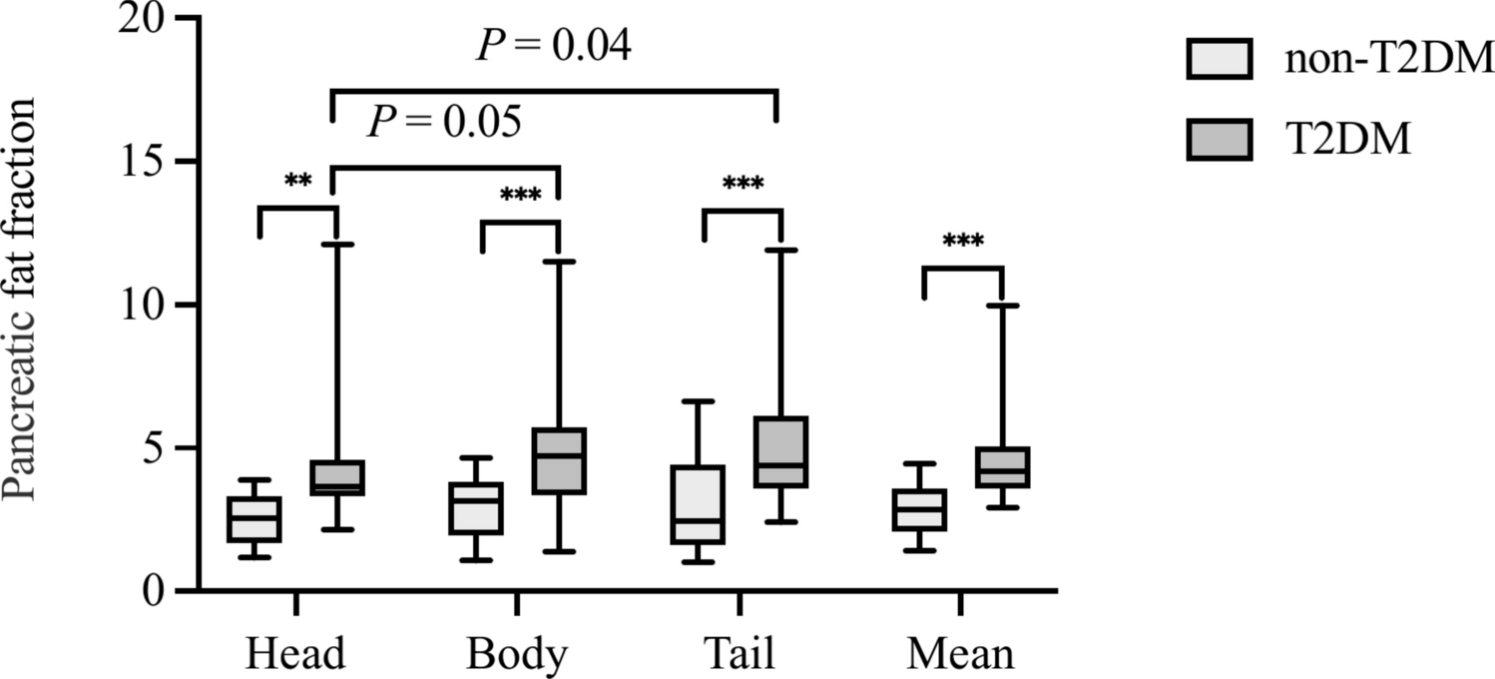
The PFF of the mean, head, body, and tail in the new-onset T2DM group compared with the non-T2DM group. A *P-*value < 0.05 indicates statistically significant difference

The PFF, LFF, and HOMA-IR index were found to be higher in the T2DM group than the control group (*P* < 0.001, 0.04, and 0.003), as shown in Table 1, Figs. 1a, b, and 2. Additionally, there was a significant difference between the T2DM and control groups in the fat fraction in the pancreatic head (*P* = 0.006), body (*P* < 0.001), tail (*P* < 0.001), and mean (*P* < 0.001). In addition, in the T2DM group, the pancreatic body and tail had marginally higher LFF compared to the head (*P* = 0.04 and 0.05), but there were no differences between the body and tail. However, there was no statistically significant difference between the three regions in the two groups.

**Fig. 3:**
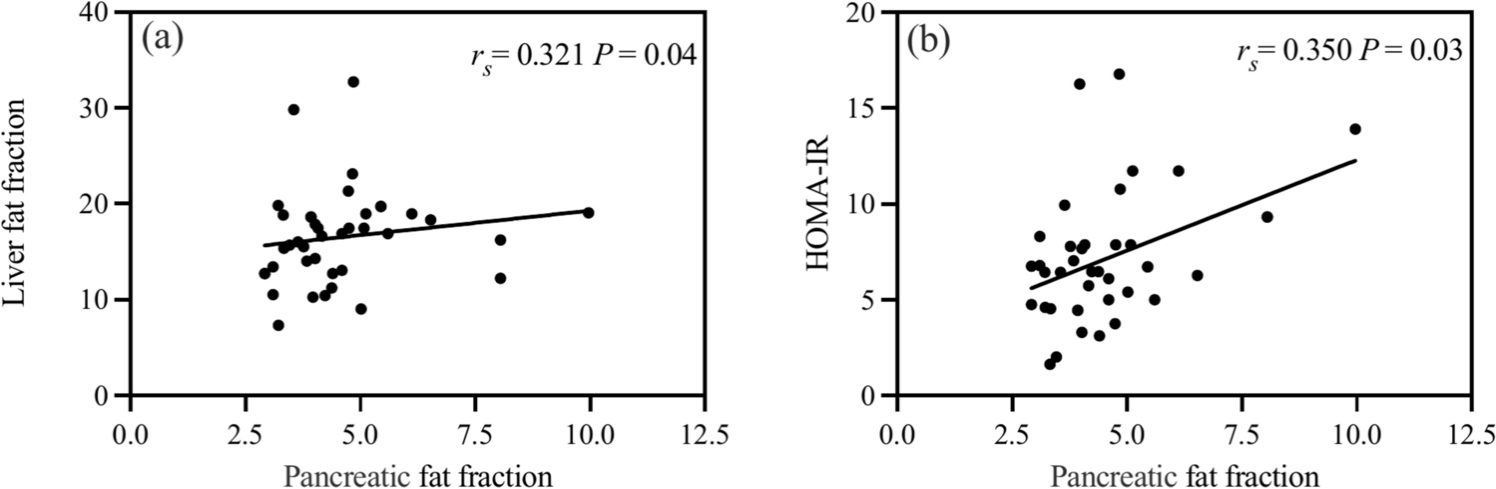
Linear correlations between the PFF and LFF (**a**), HOMA-IR (**b**) in the new-onset T2DM group. A *P*-value < 0.05 indicates statistically significant difference

As shown in Fig. 3a, b, the bivariate correlation analysis of the T2DM group revealed that the PFF was positively associated with LFF (*r*_*s*_ = 0.321, *P* = 0.04) and HOMA-IR (*r*_*s*_ = 0.350, *P* = 0.03).

### Associations between the PFF and metabolic variables in MAFLD patients with new-onset T2DM

The binary logistic regression analysis data shown in Table 2 reveal the association between PFF and the presence of new-onset T2DM in patients with MAFLD. In the unadjusted crude univariate logistic regression analysis of model 1, PFF indicated an increased risk of T2DM (*P* < 0.001 for trend). After adjustment for sex, age, BMI (model 2) and further adjustment for ALP, SUA, HOMA-IR index, and LFF (model 3), it remained positively associated with the presence of T2DM, and the association between PFF and the presence of T2DM in MAFLD patients remained stable (all *P* < 0.001 for trend).

**Table 2:** Associations between the PFF and metabolic variables in new-onset T2DM in MAFLD patients

	B statistic	OR	95% CI	*P*-value
Model 1	2.12	8.30	3.15–21.87	< 0.001
Model 2	2.39	10.88	3.57–33.20	< 0.001
Model 3	2.88	17.79	4.00–79.21	< 0.001

Binary logistic regression was used to determine the associations

Model 1: unadjusted crude model

Model 2: adjusted for sex, age, and BMI

Model 3: further adjusted for AKP, SUA, LFF, and HOMA-IR

A *P-*value < 0.05 indicates statistically significant difference

*CI* confidence interval, *OR* odds ratio

### Accuracy of the PFF and LFF in predicting new-onset T2DM in MAFLD patients

**Fig. 4:**
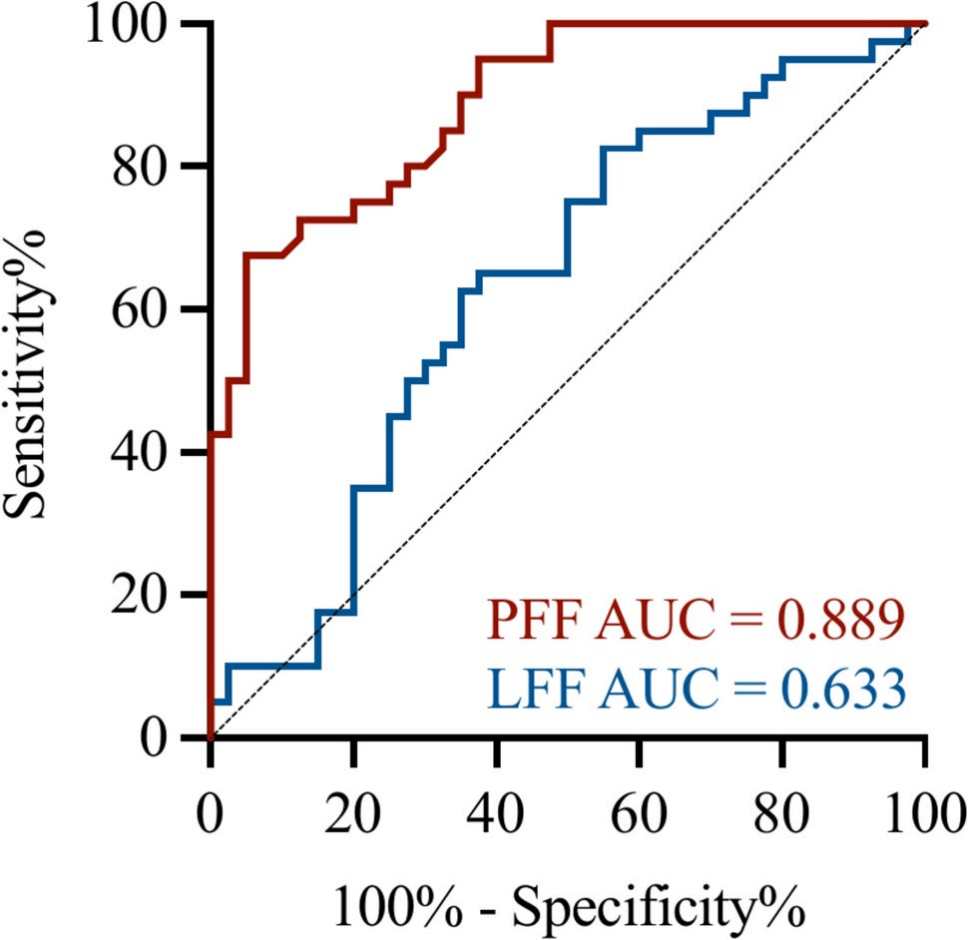
ROC curve diagnostic accuracy of the PFF and LFF used to predict new-onset T2DM in MAFLD patients. A *P*-value < 0.05 indicates statistically significant difference

As shown in Fig. 4, the AUC for PFF to identify T2DM was 0.889 (95% CI 0.822–0.957) with a sensitivity and specificity of 67.5 and 95%, respectively, using a cut-off value of 3.925%; the AUC for LFF to identify T2DM was 0.633 (95% CI 0.51–0.757) with a sensitivity and specificity of 82.5 and 55%, respectively, using a cut-off value of 12.605%.

## Discussion

This retrospective study showed that MAFLD patients with new-onset T2DM had higher PFF, LFF, and metabolic variables related to glucose and pancreatic islet cell levels than non-T2DM patients. The PFF was positively correlated with LFF and HOMA-IR. After adjustment for metabolic variables, the PFF remained an independent risk factor for incident T2DM in MAFLD patients and had a high predictive and diagnostic value for T2DM.

MRI is the most naturally fit imaging modality for the noninvasive quantification of the PFF in humans and avoids the observer variability of ultrasound and ionizing radiation of CT. It has the advantage of mapping quantitative data across an entire imaged volume and shorter acquisition times, which allows for probing fat distribution within the liver, pancreas, adjacent abdominal viscera, and more distant tissue at the same time [16–18].

The prevalence of MAFLD and T2DM has increased exponentially as a result of the global obesity pandemic. The high prevalence of T2DM is 22.5% in those with MAFLD and 43.6% in those with a more advanced form of MAFLD, such as nonalcoholic steatohepatitis (NASH) [20] due to the similar pathobiology of both conditions as a consequence of the metabolic syndrome. IR is a hallmark of T2DM and a major driver of MAFLD [21, 22]. Hepatic IR impairs the suppression of gluconeogenesis. In T2DM patients, systemic IR promotes the excessive release of FFAs from adipose tissue, which is taken up by the liver, leading to hepatic steatosis, creating a vicious cycle that accelerates liver injury. Hyperglycemia and IR not only activate pro-inflammatory pathways, exacerbate hepatocyte injury, and promote hepatic stellate cell activation, thereby driving fibrosis progression, but also lead to gut microbiota dysbiosis, characterized by reduced microbial diversity and increased intestinal permeability, further amplifying inflammation and fibrosis [23]. These patients often show more severe hepatic steatosis, inflammation, and fibrosis compared to non-diabetic MAFLD patients. The coexistence of MAFLD and T2DM is a serious health threat and increases the risk of poor prognosis and progression of other individual diseases, including cardiovascular disease, hepatocellular carcinoma, and all-cause mortality in these patients [24]. Therefore, our study focused on risk factors for new-onset T2DM in patients with MAFLD. Consistent with previous studies [25], our study revealed that in patients with MAFLD, newly diagnosed T2DM patients had higher liver fat content than non-T2DM patients, suggesting that T2DM is associated with increased fat accumulation in the liver. Therefore, predicting and assessing the risk of T2DM, especially in MAFLD patients, is essential to prevent and reduce the damage caused by T2DM.

Several studies have shown that T2DM is associated with excess intra-abdominal fat, liver fat, pancreatic fat, and ectopic fat deposition [3, 7, 9, 25]. T2DM often coexists with pancreatic fat, but most studies have focused on populations with predominantly diabetic patients, less so on those with MALFD. Although van Geenen et al. [26] pioneered the finding of histopathological evidence linking hepatic and pancreatic steatosis in postmortem specimens, their reliance on localized tissue biopsies and omission of dynamic metabolic parameters limited the generalizability of their findings to clinical populations. Our study addresses these limitations by performing whole-organ quantitative MRI mapping of PFF and LFF, circumventing sampling bias inherent to single-site histopathology, integrating HOMA-IR with imaging biomarkers, and focusing the investigation on MAFLD cohorts, a population with distinct metabolic risk profiles. Our results both confirm and refine previous findings [25–27]. The PFF-LFF linear positive correlation (*r*_*s*_ = 0.321, *P* = 0.04) in MAFLD patients with new-onset T2DM extends the pathological correlations reported by van Geenen et al. to clinical patients, while revealing at the same time that PFF is an independent risk factor for new-onset T2DM in MAFLD patients. We also found that HOMA-IR, a central mechanism of MAFLD and T2DM, was linearly and positively correlated with the PFF (*r*_*s*_ = 0.350, *P* = 0.03), suggesting a close relationship between IR and pancreatic fat as a possible pathogenesis mechanism. This risk was undetectable by conventional histology. Thus, our findings indicated the diagnostic value of PFF and LFF in new-onset T2DM. We found that they were good potential radiological biomarkers to help clinicians screen high-risk individuals for T2DM in MAFLD. Notably, our PFF threshold of 3.925% (AUC = 0.889) differs significantly from the 6.2% cutoff derived from mixed populations by Singh et al. [28], suggesting that concurrent hepatic steatosis potentiates pancreatic lipotoxicity at lower fat thresholds and is more likely to induce T2DM and adverse outcomes.

Several studies have found no significant correlation between the PFF and T2DM [13–15], but their findings must be interpreted with caution due to methodological and conceptual limitations. There may be several reasons for these conflicting findings. First, the study population was relatively small and lacked diversity in terms of T2DM severity and duration. Second, the analysis did not account for potential confounders, such as IR, genetic predisposition, or lifestyle factors, which may affect T2DM [15, 29]. Moreover, pancreatic fat may exert its effects indirectly through mechanisms like lipotoxicity, inflammation, or β-cell dysfunction. Some studies have demonstrated that pancreatic fat is associated with β-cell dysfunction, a hallmark of T2DM, suggesting that the relationship may be more complex than a simple correlation [30–32]. Third, the heterogeneity in imaging techniques and quantification methods. Studies using CT or MRI may differ in sensitivity, specificity, or sequence for detecting pancreatic fat, leading to inconsistent results. Lastly, the distribution of pancreatic fat is also controversial. The distribution of pancreatic fat deposits can be uneven, and focal pancreatic fat deposits typically occur in the tail and anterior head of the pancreas [33, 34]. However, in previous studies, the fat content of each part of the pancreas was found to be uniform [35, 36]. Although Chai et al. [35] found no significant difference in the distribution of heterotopic fat deposition in the three regions of the pancreas, the fat content of the pancreatic head was significantly higher in T2DM compared to the body and tail. The present study found that the distribution of pancreatic fat in the head, body, and tail regions of the pancreas was similar in the two groups, with no statistically significant difference between the three regions. However, the fat content of the body and tail of the pancreas was slightly higher compared to the head of the pancreas in the T2DM group.

However, this study has several limitations. First, due to the lack of relevant data for the oral glucose tolerance test (OGTT), we were unable to calculate the disposition index and accurately assess β-cell function in the context of IR for further investigation. Second, since the mechanisms of ectopic fat in the pancreas are unclear, this MRI study could not accurately determine its complex pathological influence. Third, due to the retrospective nature of this study, causality could not be inferred. Future research should focus on standardizing imaging protocols, expanding sample sizes, and establishing longitudinal or animal studies to investigate the temporal relationship between ectopic fat deposition and metabolic outcomes, including IR, β-cell dysfunction, and disease progression. The integration of genomic and metabolomic multi-omics approaches may reveal mechanistic links between ectopic fat accumulation and T2DM pathogenesis of insulin secretion, inflammation, and oxidative stress.

In conclusion, this study revealed that T2DM is associated with increased LFF and PFF in patients with MAFLD. After adjustment for various metabolic factors, the PFF remained an independent risk factor for T2DM and played a prominent role in incident T2DM with high predictive and diagnostic value. The PFF based on MRI has the advantages of simplicity, stability, and reproducibility, and could be a promising radiological biomarker to help clinicians in the prevention, diagnosis, and therapeutic evaluation of T2DM.
